# Characterization and genetic analysis of extensively drug-resistant hospital acquired* Pseudomonas aeruginosa* isolates

**DOI:** 10.1186/s12866-024-03321-5

**Published:** 2024-06-26

**Authors:** Mai A. Abdelaziz, Abeer M. Abd El-Aziz, Mohamed M. A. El-Sokkary, Rasha Barwa

**Affiliations:** Department of Microbiology and Immunology, Faculty of Pharmacy, Mansoura University, Mansoura, Egypt

**Keywords:** *Pseudomonas aeruginosa*, Hospital-acquired infections, Extensively drug-resistant, ERIC-PCR

## Abstract

**Background:**

The incidence of hospital-acquired infections in extensively drug-resistant *Pseudomonas aeruginosa* (XDR-PA) has been increasing worldwide and is frequently associated with an increase in mortality and morbidity rates. The aim of this study was to characterize clinical XDR-PA isolates recovered during six months at three different hospitals in Egypt.

**Results:**

Seventy hospital-acquired clinical isolates of *P. aeruginosa* were classified into multidrug-resistant (MDR), extensively drug-resistant (XDR) and pandrug-resistant (PDR), according to their antimicrobial resistance profile. In addition, the possession of genes associated with mobile genetic elements and genes encoding antimicrobial resistance determinants among isolates were detected using polymerase chain reaction. As a result, a significant percentage of the isolates (75.7%) were XDR, while 18.5% were MDR, however only 5.7% of the isolates were non-MDR. The phenotypic detection of carbapenemases, extended-spectrum β-lactamases (ESBLs) and metallo β-lactamase (MBL) enzymes showed that 73.6% of XDR-PA isolates were carbapenemases producers, whereas 75.5% and 88.7% of XDR-PA isolates produced ESBLs and MBL respectively. In addition, PCR screening showed that *oxa* gene was the most frequently detected gene of carbapenemases (91.4%), while *aac(6ʹ)-lb* gene was mostly detected (84.3%) among the screened aminoglycosides-resistance genes. Furthermore, the molecular detection of the colistin resistance gene showed that 12.9% of isolates harbored *mcr-1* gene. Concerning mobile genetic element markers (*intI, traA, tnp513, and merA), intI* was the highest detected gene as it was amplified in 67 isolates (95.7%). Finally*,* phylogenetic and molecular typing of the isolates via ERIC-PCR analysis revealed 10 different ERIC fingerprints.

**Conclusion:**

The present study revealed a high prevalence of XDR-PA in hospital settings which were resistant to a variety of antibiotics due to several mechanisms. In addition, 98% of the XDR-PA clinical isolates contained at least one gene associated with movable genetic elements, which could have aided the evolution of these XDR-PA strains. To reduce spread of drug resistance, judicious use of antimicrobial agents and strict infection control measures are therefore essential.

**Supplementary Information:**

The online version contains supplementary material available at 10.1186/s12866-024-03321-5.

## Introduction

*Pseudomonas aeruginosa* is an opportunistic worldwide pathogen responsible for a wide range of bacterial complications, including pneumonia, endocarditis, urinary tract infections, burn and wound infections, in addition to, sepsis and bacteremia [1, 2]. This superbug has been reported to be one of the most prevalent microbes isolated from hospitalized patients, accounting for 10% of all nosocomial infections and ranking in the top three most often reported hospital-acquired pathogens [3].

*P. aeruginosa* is armed with a diverse arsenal of antimicrobial resistance mechanisms, where it can rapidly develop resistance to different antimicrobial agents by acquiring of resistance genes in movable genetic elements [1]. According to the International Nosocomial Infection Control Consortium (INICC) surveillance study, by analyzing the antimicrobial sensitivity pattern of strains obtained from patients admitted to intensive care units conducted in 45 countries, approximately 50% of *P. aeruginosa* infections were resistant to recently discovered and potent antibiotics [4]. Therefore, the rise of multidrug-resistant (MDR), extensively drug-resistant (XDR), and pandrug-resistant (PDR) *P.aeruginosa* becomes an emerging public health threat [5]. Since the use of antibiotics as the first line of treatment for bacterial infections, the emergence of XDR bacteria has raised concerns about a post-antibiotic age, in which many bacterial infections may be untreatable with conventional antibiotics [6].

Extensively drug-resistant *Pseudomonas aeruginosa* (XDR-PA) is defined as insusceptibility of the isolate to at least one antibiotic in all, but two or more antimicrobial categories [7–9]. Based on emerging studies, antibiotic resistance of XDR-PA is usually associated with the production of different β-lactamases, colistin resistance enzymes, aminoglycoside-modifying enzymes (AMEs), expression of outer membrane protein, and active efflux system. Furthermore, integron-carrying drug-resistant genes and bacterial biofilm production are frequently linked to elevated antibiotic resistance in XDR-PA strains [1, 10].

The incidence of hospital-acquired infections in XDR-PA has been increasing in Egypt and worldwide [11, 12]. However, there is a lack of sufficient recent studies addressing this issue in our country. Therefore, the present work aimed to characterize and determine the prevalence of XDR-PA clinical isolates associated with nosocomial infections in our country. Additionally, we aimed to investigate the various mechanisms involved in their antimicrobial resistance. The ultimate goal of this study is to develop urgent and efficient extensively drug-resistant overcoming strategies in hospitals and to prevent the emergence of pandrug-resistant *P. aeruginosa* strains in our region.

## Methodology

### Bacterial isolates

An initial collection of 125 clinical specimens were obtained from inpatients at different hospitals in Egypt in the time between September 2019 and March 2020. The protocol used in this study adheres to the ethical guidelines and the principles of handling human subjects in medical research adopted by ‘The Research Ethics Committee of the Faculty of Pharmacy at Mansoura University, Egypt, which is in accordance with the Code of Ethics of the World Medical Association (Declaration of Helsinki regarding the involvement of human subjects). The study’s protocol was approved by the committee with the ethical codes 2019–110 and 2023–16. Specimens were obtained from various clinical sources (burns, wounds, urine, sputum, blood, and pus) at three different hospitals; hospital 1 (H1), hospital 2 (H2), and hospital 3 (H3). The three hospitals selected in our study, represents large central hospitals, located in different areas in Cairo and Mansoura. These hospitals offer a wide range of medical specialties and serve patients from all over the country, which allowed us to have diverse samples and avoid limitations associated with studying only one hospital or city. Only one isolate per patient was used in the study to avoid over representation of certain strains. Seventy isolates were identified as *Pseudomonas aeruginosa* by streaking on cetrimide agar plates, which were then incubated for 24 h at 37^◦^C. The resulting colonies were examined microscopically after Gram staining and identified biochemically as being positive oxidase and catalase producers, citrate utilizers and nitrate reducers. Additionally, they exhibited a characteristic sweetish odor and produced a blue-green pigment (pyocyanin) on the cetrimide agar plates. *P. aeruginosa* isolates were further confirmed by molecular identification of the *opr*L gene specific for *P. aeruginosa* using the primer pair listed in Table 1. In case of H1 hospital*,* a total of 52 isolates (74.3% of total isolates) were identified, including 13 burn isolates (b), 14 wound isolates (w), 16 urine isolates (u), 6 sputum isolates (s), 2 blood isolates (bl), and one isolate taken from pus (p). In case of H2*,* a total of 15 (21.4%) isolates were identified including 11 isolates, obtained from burns (b), 3 isolates obtained from wounds (w) and one isolate taken from pus) p). In case of H3 hospital*,* a total of 3 (4.3%) isolates were identified from burn specimens (b). For easier identification, all isolates were coded based on the hospitals they were obtained from (H1, H2, and H3), in addition to the clinical sources: burn (b), wound (w), urine (u), sputum (s), blood (bl), and pus (p). Finally, seventy isolates were cultivated in Luria–Bertani (LB) medium at 37^◦^C and preserved in 25% (v/v) glycerol at—80^◦^C until further analysis.

**Table 1:** Primers sequencesused for screening the tested genes

Type of Gene	Target gene	Primer type	Nucleotide sequence (5′-3′)	Annealing temperature (°C)	Amplicon size (bp)	References
**Identification gene**	***opr*L**	*opr*L -F	CGAGTACAACATGGCTCTGG	**53**	**116**	[13]
		*opr*L -R	ACCGGACGCTCTTTACCATA			
**Carbapenemase- encoding genes**	***ndm-1***	ndm-1-F	ACTTCCTATCTCGACATGC	**52**	**133**	[14]
		ndm-1-R	TGATCCAGTTGAGGATCTG			
	***vim-1***	vim-1-F	TGTTATGGAGCAGCAACGATG	**56**	**920**	[14]
		vim-1-R	AAAGTCCCGCTCCAACGATT			
	***vim-2***	vim-2-F	GTCTATTTGACCGCGTCTATC	**55**	**774**	[15]
		vim-2-R	CTACTCAACGACTGAGCGAT			
	***oxa***	oxa-F	AAGTGTGCAACGCAAATGGC	**55**	**137**	[15]
		oxa-R	CTGTTCCAGATCTCCATTCC			
**Aminoglycosidesresistance genes**	***aac(6ʹ)-lb***	aac(6ʹ)-lb-F	TTGCGATGCTCTATGAGTGG	**55**	**481**	[16]
		aac(6ʹ)-lb-R	CTCGAATGCCTGGCGTGTTT			
	***aac(3)-II***	aac(3)-II-F	GCGGAAGGCAATAACGGAG	**54**	**567**	This study, [17]
		aac(3)-II-R	CCAAGCATCGGCATCTCATA			
	***rmtB***	rmtB-F	GCTTTCTGCGGGCGATGTAA	**53**	**173**	[18]
		rmtB-R	ATGCAATGCCGCGCTCGTAT			
	***aph(3')-I***	aph(3ˋ)I-F	TTATGCCTCTTCCGACCATC	**53**	**222**	[17]
		aph(3ˋ)I-R	GCCTGAGCGAGACGAAATAC			
**Colistin resistance gene**	***mcr-1***	mcr-1-F	AGTCCGTTTGTTCTTGTGGC	**58**	**320**	[19]
		mcr-1-R	AGATCCTTGGTCTCGGCTTG			
**Genetic marker of integron**	***intI***	intI- F	CCGAGGATGCGAACCACTTC	**53**	**373**	[20]
		intI- R	CCGCCACTGCGCCGTTACCA			
**Genetic markers of transposon**	***merA***	merA-F	GACCAGCCGCAGTTCGTCTA	**62**	**462**	[20]
		merA-R	GCAGCASGAAAGCTGCTTCA			
	***tnp513***	tnp513-F	ATGTCGCTGGCAAGGAACGC	**64**	**200**	[1]
		tnp513-R	GGGTTCGCTGCGAGGATTGT			
**Genetic marker of plasmid**	***traA***	traA-F	AAGTGTTCAGGGTGCTTCTGCGC	**43**	**310**	[1]
		traA-R	GTCATGTACATGATGACCATTT			
**Typing gene** **ERIC-PCR**		ERIC1	ATGTAAGCTCCTGGGGATTCAC	**48**	**variable**	[21]
		ERIC2	AAGTAAGTGACTGGGGTGAGCG			

*F* Forward, *R* Reverse, *bp* Base pair

### Antimicrobial susceptibility testing for isolates

Kirby-Bauer disk diffusion method was used to identify the susceptibility of all 70 isolates to different antimicrobials using Mueller–Hinton agar plates [22]. The susceptibility pattern was determined for 16 different antimicrobials, of eight different classes including antipseudomonal carbapenems, antipseudomonal cephalosporins, aminoglycosides, antipseudomonal penicillin with β-lactamase inhibitor, monobactams, antipseudomonal fluoroquinolones, phosphonic acids, and polymyxins. In this study, the following antibiotic disks (Bioanalyse®, Turkey) were used: Gentamicin (10 μg), Tobramycin (10 μg), Amikacin (30 μg), Netilmicin (30 μg), Imipenem (10 μg), Meropenem (10 μg), Doripenem (10 μg), Ceftazidime(30 μg), Cefepime (30 μg), Ciprofloxacin (5 μg), Levofloxacin (5 μg), Piperacillin-tazobactam (100/10 μg), Aztreonam (30 μg) and Fosfomycin (200 μg). However, the susceptibilities of different isolates against colistin (colistin-sulfate, Sigma-Aldrich, Germany) and polymyxin B (Titan Media, India) were determined by broth micro-dilution method, as recommended by Clinical and Laboratory Standard Institute (CLSI) guidelines. As study controls, two standard *P. aeruginosa* strains PAO1 and PA14 were used as reference strains for antibiotic susceptibility testing. Bacterial isolates were classified as resistant, intermediate, or susceptible, according to the guidelines indicated by both CLSI 2020 [23] and European Committee on Antimicrobial Susceptibility Testing (EUCAST, 2021) [24]. Bacterial isolates were further classified into MDR, XDR, and PDR, according to the International standard definitions for acquired resistance. These definitions were established through a joint initiative by the European Centre for Disease Prevention and Control (ECDC) and the Centers for Disease Control and Prevention (CDC) [7–9]. Multiple antibiotic resistance (MAR) index was calculated for each isolate as the ratio between the number of antimicrobial agents to which the isolate showed resistance and the total number of antimicrobial agents to which the isolate had been assessed for susceptibility [25]. A MAR index > 0.2 indicates a ‘high-risk’ source of contamination, where antimicrobials are overused. For more information concerning strains relatedness, the unweighted pair group method with arithmetic mean clustering method (UPGMA) software was used to construct antibiotic resistance profiles dendrogram [26].

### Phenotypic detection of carbapenemases, extended-spectrum β-lactamase (ESBL) and metallo β-lactamase (MBL) enzymes production

The modified Hodge test (MHT) for screening of carbapenemase production was applied to 53 XDR-PA isolates, as previously described before [15, 27–29] using meropenem disk (10 μg), in presence of an indicator strain. In this study, PAO1 was used as a negative control, while H1u16 clinical isolate (harboring all tested resistant genes) was used as a positive control. For results interpretation, a positive MHT was indicated by the presence of a clover-leaf-shaped or a distorted inhibition zone [30]. For ESBL detection, the combined disc diffusion test was performed. The tested strains were inoculated on Mueller Hinton agar plates and screened by disks of ceftazidime (CAZ-30 μg) and ceftazidime, in combination with clavulanic acid (30 μg/10 μg). For interpretation of the results, ≥ 5 mm difference between the zone of inhibition of the disk containing clavulanate, compared to the effect of antibiotic disk alone was considered as an ESBL-positive isolate [31, 32]. The test was performed for all XDR-PA isolates, in addition to negative and positive control strains. In case of MBL detection, combined disc test (CDT) was performed on the test organism inoculated onto the surface of Mueller Hinton agar plate by using two Imipenem (IMP-10 μg) disks. In detection with EDTA, ten microliters of 0.5 M EDTA solution (pH 8) were applied to one of the IMP discs. All XDR-PA isolates and control strains were subjected to the assay. The inhibitory zones of the IMP discs with and without EDTA were compared following an overnight incubation at 37 °C. As previously indicated, values more than 7 mm increase in the inhibition zone diameter for the imipenem disc in the presence of EDTA was considered as a positive test result [33].

### Molecular detection of *P*. *aeruginosa* resistance genes and genes associated with mobile genetic elements

The possession of antimicrobial resistance genes by *P.aeruginosa* isolates was investigated by PCR using the oligonucleotide primers listed in Table 1. PCR screening targeted the detection of carbapenem resistance genes (*ndm-1, vim-1, vim-2* and *oxa****),*** four aminoglycosides resistance genes (*aac (3)-II, aac(6ʹ)-lb,aph(3')-I,* and *rmtB*) and the colistin resistance gene *mcr-1*. For each isolate, any DNA template for PCR was prepared by heating three to six pure colonies suspended in 100 μl of nuclease free water at 95˚C for 10 min, followed by 10 min centrifugation at 10000 xg to remove cellular debris. Using the following mixture, the PCR reaction was adjusted to a total volume of 25 μl:12.5 μl of ready-to-use Dream Taq™ Green PCR Master Mix [2x] (Thermo Scientific, US), 2 μl of target DNA,1 μl of forward primer (10 μM), 1 μl of reverse primer (10 μM), and 8.5 μl of nuclease-free water. Additionally, a negative control without a DNA template was also used. Thermo-cycling settings involved an initial denaturation step at 94 °C for 5 min, followed by 35 cycles each containing three steps: denaturation at 94 °C for 30 s, annealing at temperatures specified in Table 1 for each primer pair for 30 s, extension at 72 °C for 1 min, and finally an extension step at 72˚C for 7 min. Agarose gel electrophoresis using 1.5% agarose gels was used to analyze the PCR products, followed by ethidium bromide staining to visualize the gels under UV radiation. The amplicons produced were compared with GeneRuler 100 bp plus (Thermo Fisher Scientific^Tm^,UK) DNA marker. Similarly, PCR was also carried out to detect the possession of different mobile genetic element markers including integron (*intI)* gene*,* transposon *(tnp51 and merA)* genes and plasmid *(traA)* gene.

### Molecular typing by Enterobacterial repetitive intergenic consensus PCR (ERIC-PCR)

Using oligonucleotide primers (ERIC-1 and ERIC-2), listed in Table 1, molecular genotyping of seventy *P. aeruginosa* isolates was performed by ERIC-PCR assay [21]. For each PCR reaction, amplification program started with an initial denaturation of DNA at 95°C for 5 min, followed by 35 cycles of denaturation at 95°C for 40 s, annealing at 48°C for 1 min, and extension at 72°C for 1.5 min. After a final elongation step at 72 °C for 7 min, the resulting patterns obtained were visualized under UV and the results interpreted using GelJ ® software [34]. A similarity matrix, based on Pearson correlation (optimization 1%; position tolerance 1%), was calculated, and the corresponding dendrogram was constructed according to their similarities using the unweighted pair group method with arithmetic averages (UPGMA) [35, 36].

### Statistical analysis and data interpretation

Data was analyzed using SPSS software, version 25 (SPSS Inc., PASW statistics for windows version 25. Chicago: SPSS Inc.). Qualitative data were described using numbers and percentages. The significance of the results was judged at the (≤ 0.05) level. Chi-Squareand Monte Carlo tests were used to compare qualitative data between groups (XDR, MDR and non-MDR) and groups (H1, H2 and H3) as appropriate.

## Results

### Isolation and identification of the isolates

A total of 70 isolates were identified as *P. aeruginosa*, where the selected isolates were all associated with hospital-acquired infections, which was not present during the time of admission but occurring during the process of receiving health care after 48 h of hospital admission. The *oprL* gene was amplified in all *P. aeruginosa* isolates confirming their identification (Table S1).

### Antimicrobial susceptibility testing and resistance pattern of the isolated *P*. *aeruginosa*

**Fig. 1:**
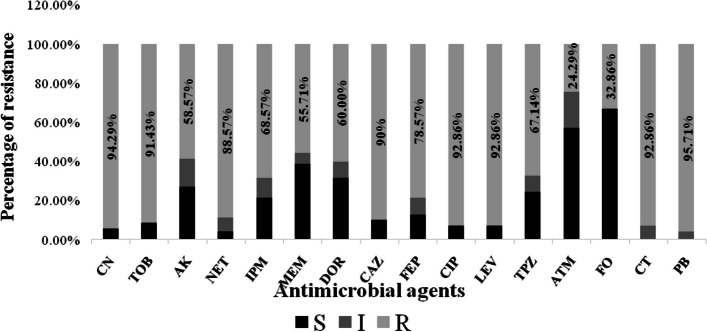
Resistance percentage of *P. aeruginosa* isolates to different antimicrobials. CN:gentamicin, TOB: tobramycin, AK: amikacin, NET: netilmicin, IPM: imipenem, MEM: meropenem, DOR: doripenem, CAZ: ceftazidime, FEP: cefepime, CIP: ciprofloxacin, LEV: levofloxacin, TPZ: piperacillin-tazobactam, ATM: aztreonam, FO: fosfomycin, CT: colistin-sulfate, PB: polymyxin B, S: sensitive, I: intermediate, R: resistant

The antimicrobial susceptibility testing for the seventy isolates showed a high frequency of resistance against gentamicin (94.29%), ciprofloxacin (92.86%) and levofloxacin (92.86%). In contrast, the lowest frequencies of resistance were observed toward aztreonam (24.29%). However, intermediate levels of resistance were detected for fosfomycin (32.86%) and meropenem (55.71%). Moreover, it was found that 92.86% and 95.71% of the isolates were resistant to colistin-sulfate and polymyxin B respectively (Fig. 1).

**Fig. 2:**
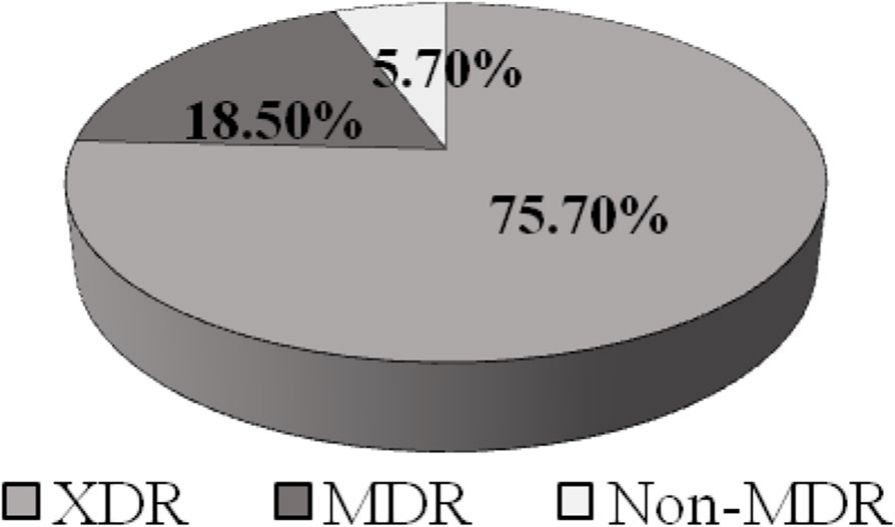
Percentage of different resistance categories among total clinical isolates of *P.aeruginosa* XDR: Extensively-drug resistant, MDR:Multidrug-resistant, Non-MDR: Non- multidrug resistant

Out of the total isolates, 53 (75.7%), 13 (18.5%), and 4 (5.7%) were classified as XDR, MDR, and non-MDR, respectively. However, no pandrug-resistant isolates were detected (Fig. 2). The differences in the distribution of XDR, MDR, and non-MDR isolates among the different clinical sources are not statistically significant, as indicated by the *P*-values for the Monte Carlo and chi-square tests (all *P*-values > 0.05) (Table 2). As demonstrated in Table 3, there is no significant difference in the proportions of XDR, compared to MDR and non-MDR isolates in each clinical source for hospital H1. However, in H2, there is a significant difference in the proportion of XDR compared to MDR and non-MDR isolates in the burn clinical source (*P* = 0.036). Interestingly, concerning H3, a significant *P*-value 0.0001 is obtained, which indicates a remarkable difference between the proportion of non-MDR, compared to XDR and MDR in burn specimens.

**Table 2:** Distribution of XDR, MDR and Non-MDR samples among *Pseudomonas aeruginosa* clinical isolates obtained from different clinical sources

Clinical source	Total number of isolates	XDR isolates no. (%)	MDR isolates no. (%)	Non-MDR (Sensitive) isolates no. (%)	χ^2^/MC	*P* value
Burn	27	17 (63%)	8 (29.6%)	2 (7.4%)	4.06	0.131
Wound	17	15 (88.2%)	1 (5.9%)	1 (5.9%)	2.41	0.299
Urine	16	15 (93.8%)	0 (0%)	1 (6.3%)	4.75	0.09
Sputum	6	3 (50%)	3 (50%)	0 (0%)	4.43	0.109
Blood	2	2 (100%)	0 (0%)	0 (0%)	0.66	0.719
Pus	2	1 (50%)	1 (50%)	0 (0%)	1.39	0.498
**Total no. of isolates**	70	53 (75.7%)	13 (18.6%)	4 (5.7%)		

*XDR* Extensively-drug resistant, *MDR* Multidrug-resistant, *Non-MDR* Non- multidrug resistant, *MC* Monte Carlo test, χ^2^ Chi-Square test, *P* probability

**Table 3:** Distribution of XDR, MDR and Non-MDR samples among *P.aeruginosa* clinical isolates obtained from different clinical sources and different hospitals

Hospital	Clinical source	Number of isolates	XDR no *N* = 53	MDR no *N* = 13	Non-MDR no *N* = 4	χ^2/MC^	*P* value
**H1**	burn	13	10	3	0	1.09	0.579
	wound	14	13	1	0	2.91	0.233
	urine	16	15	0	1	4.75	0.093
	sputum	6	3	3	0	4.44	0.109
	blood	2	2	0	0	0.66	0.719
	pus	1	0	1	0	4.45	0.108
**H2**	Burn	11	6	5	0	6.59	0.036*
	Wound	3	2	0	1	4.8	0.09
	pus	1	1	0	0	0.325	0.850
**H3**	burn	3	1	0	2	21.70	0.0001*
**Total no. of isolates**		70	53	13	4		

*XDR* Extensively-drug resistant, *MDR* Multidrug-resistant, *Non-MDR* Non- multidrug resistant, *MC* Monte Carlo test, χ^*2*^ Chi-Square test, *P* probability

(*) means significant

**Fig. 3:**
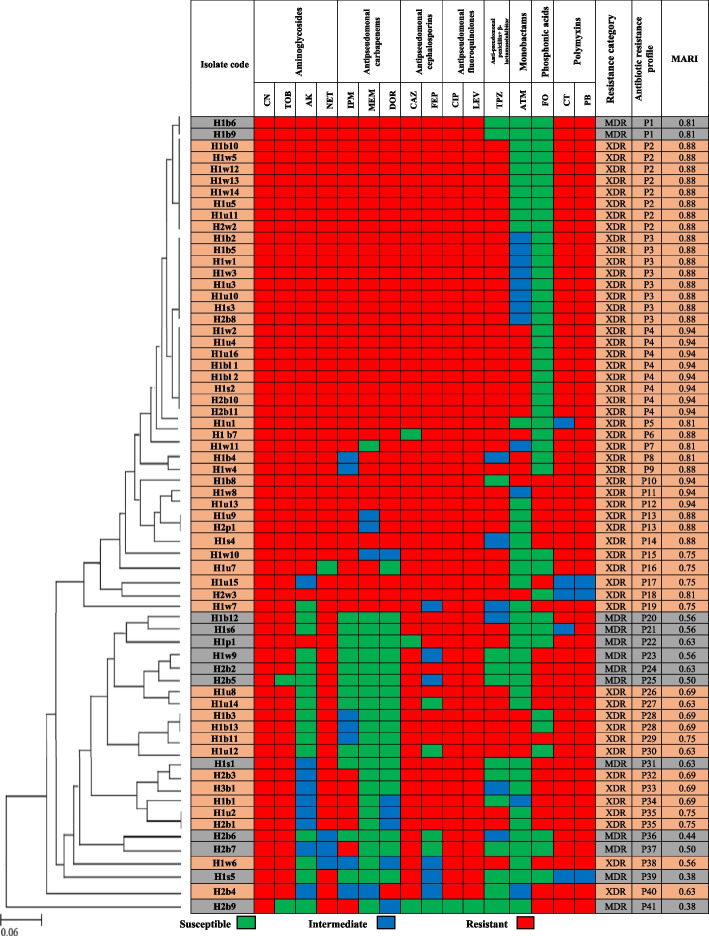
Heat map showing antibiotic resistance profiles and MARI of MDR and XDR *P.aeruginosa* isolates, MARI: Multiple antibiotic resistance index, dendrogram was constructed using UPGMA software, H1: Hospital 1, H2: Hospital 2, H3: Hospital 3, b: burn, w: wound, u: urine, bl: blood, s: sputum, p: pus

As illustrated in Fig. 3, the MDR isolates showed high resistances to gentamicin, ciprofloxacin, levofloxacin and polymyxin B antimicrobials, while all of the MDR isolates were sensitive to aztreonam antibiotic. The MARI ranged from 0.38 to 0.81, and the dendrogram showed that MDR isolates represent 12 unique antibiotic resistance profiles. Regarding XDR isolates, they showed high resistance to gentamicin, tobramycin, ciprofloxacin, levofloxacin and ceftazidime, while fosfomycin and aztreonam were the most effective antimicrobial agents. The MARI ranged from 0.56 to 0.94, and the 53 XDR isolates displayed 29 antibiotic resistance profiles, where P2 and P3 profiles each included 7 isolates from H1 and one isolate from H2, however P4 included 6 isolates from H1 and 2 isolates from H2.

### Phenotypic detection of carbapenemases, extended-spectrum β-lactamase (ESBL), and Metallo β-lactamase (MBL) enzymes production

**Fig. 4:**
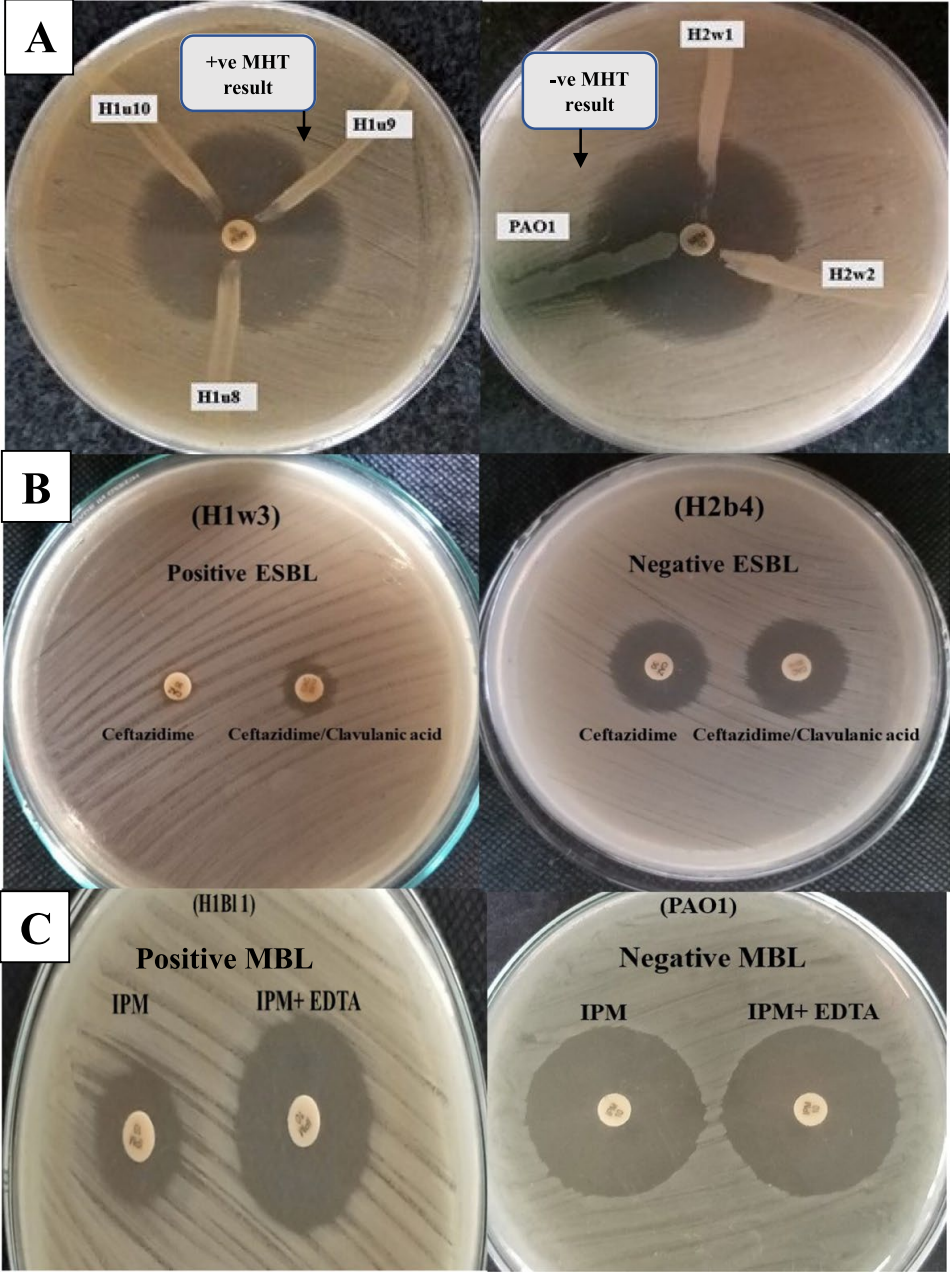
Phenotypic detection of β-lactamase production for *P. aeruginosa* clinical isolates. **A** Modified Hodge test for carbapenemase productiondetection. **B** Combined disk diffusion test for (ESBLs) productiondetection. **C** Combined disk test was for MBL detection. MHT: Modified-Hodge test, ESBL: Extended-spectrum β-lactamase, MBL: Metallo β-lactamase, IPM: Imepenim

**Fig. 5:**
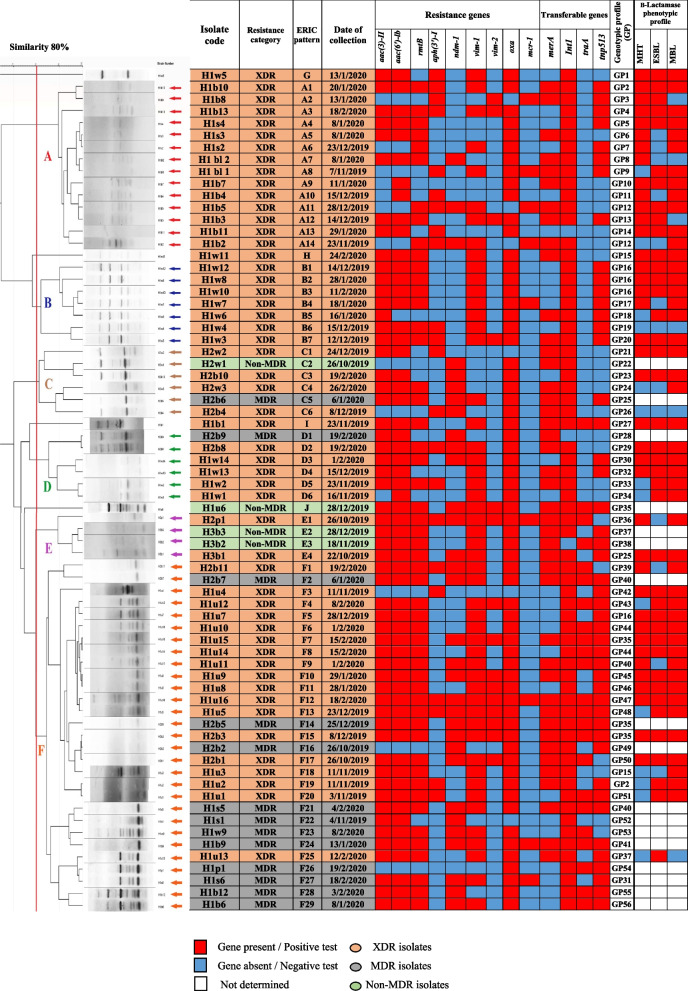
ERIC-PCR Clustering dendrogram of 70 P. aeruginosa clinical isolates at 80% similarity using UPGMA, each clinical source, date of collection, resistance category, genotypic profile and β-lactamase phenotypic profile ofthe isolates are also shown, H1: Hospital 1, H2: Hospital 2, H3: Hospital 3, b: burn, w: wound, u: urine, bl: blood, s: sputum, p: pus

Modified Hodge test (MHT) was performed on 53 XDR-PA isolates, where thirty nine isolates (73.6% of XDR) showed a clover leaf-like indentation at the point of intersection of the isolate with the indicator strain, within the inhibition zone of the meropenem disk, confirming positive carbapenemase enzyme production. On the other hand, 14 isolates (26.4% of XDR) showed negative MHT results (Figs. 4A and 5). The presence of (ESBL) enzymes was studied on XDR-PA isolates through the combined disk diffusion test indicating 40 isolates of XDR (75.5% of XDR) and considered as ESBLs producers. In contrast,13 isolates (24.5% of XDR) showed negative results (Figs. 4B and 5). The MBL enzymes detection by the combined disk test (CDT) showed that 47 isolates (88.7% of XDR) were MBL enzyme producers, while 6 isolates (11.3% of XDR) exhibited non-enzyme producing capabilities (Figs. 4C and 5).

### Molecular analysis of the strains

PCR screening showed that *oxa* was the most frequently detected gene among the screened carbapenemase-encoding genes, as it was amplified in 64 isolates (91.4%), followed by *vim-1* and *ndm-1* genes, which were amplified in 52 isolates (74.3%) and 32 isolates (45.7%) respectively. On the other hand, *vim-2* gene was the least detected gene, as it was present only in 15 isolates (21.4%) (Fig. 1A Supp.). Among the screened aminoglycosides-resistance genes, *aac(6ʹ)-lb* was the highest detected gene as it was amplified in 59 isolates (84.3%). Concerning *aac(3)-II* and *rmtB* genes, they were amplified in 57 (81.4%) and 52 isolates (74.3%) respectively, while *aph (3')-I* gene was the least detected gene, as it was detected only in 22 isolates (31.4%). Intrestingly, 52 isolates (74.3%) harboured at least three aminoglycosides-resistance genes (Fig. 1B Supp.)*.* In this study, the genetic detection of the colistin resistance *(mcr-1)* was positive for 9 isolates (12.9%)*,* where 8 of them were colistin resistant (Fig. 1C Supp.). Intrestingly, *intI* was the most predominant gene among the screened mobile genetic element markers, as it was amplified in 67 isolates (95.7%), followed by *tnp513* and *merA* genes, as they were detected in 47 (67.1%) and 43 ( 61.4%) isolates, respectively. In contrast, *traA* was the least detected gene, as it was harbored by only 23 isolates (32.9%) (Fig. 1D Supp.).

### Distribution of resistance genes among different hospitals, MDR and XDR isolates

In this study, the *oxa* gene was the most commonly detected carbapenemase-encoding gene in H1 hospital. However, in H2 hospital, both *oxa* and *ndm-1* genes were the most predominant. In case of H3 hospital, *oxa* and *vim-1* genes were the most commonly detected. Interestingly, *ndm-1* gene was detected in 93.3% of isolates from H2 hospital, which was significantly higher than in the other hospitals (*P* < 0.05, Table 4).

**Table 4:** Distribution of resistance genes among different hospitals, MDR and XDR isolates

Gene type	Gene	Number of isolates				*P* value	Number of isolates		*P* value
		**Total no of isolates *n* = 70 (%)**	**H1** ***n* = 52 (%)**	**H2** ***n* = 15 (%)**	**H3** ***n* = 3 (%)**		**MDR isolates *n* = 13 (%)**	**XDR isolates *n* = 53 (%)**	
**Carbapenemase- encoding genes**	***ndm-1***	**32 (45.7%)**	17 (32.7%)	14 (93.3%)	1 (33.3%)	0.0001*	12 (92.3%)	18 (33.9%)	0.001*
	***vim-1***	**52 (74.3%)**	41 (78.8%)	8 (53.3%)	3 (100%)	0.079	8 (61.5%)	41 (77.4%)	0.242
	***vim-2***	**15 (21.4%)**	11 (21.2%)	4 (26.7%)	0 (0%)	0.587	1 (7.7%)	13 (24.5%)	0.183
	***Oxa***	**64 (91.4%)**	47 (90.4%)	14 (93.3%)	3 (100%)	0.809	12 (92.3%)	48 (90.6%)	0.844
**Aminoglycoside resistance genes**	***aac(6ʹ)-lb***	**59 (84.3%)**	44 (84.6%)	12 (80%)	3 (100%)	0.679	11 (84.6%)	45 (84.9%)	0.979
	***aac(3)-II***	**57 (81.4%)**	42 (80.8%)	12 (80%)	3 (100%)	0.698	11 (84.6%)	43 (81.1%)	0.770
	***rmtB***	**52 (74.3%)**	40 (76.9%)	9 (60%)	3 (100%)	0.242	9 (69.2%)	40 (75.5%)	0.645
	***aph(3')-I***	**22 (31.4%)**	17 (32.7%)	5 (33.3%)	0 (0%)	0.486	0 (0%)	22 (41.5%)	0.004*
**Colistin resistance gene**	***mcr-1***	**9 (12.9%)**	8 (15.4%)	1 (6.7%)	0 (0%)	0.535	2 (15.4%)	7 (13.2%)	0.165

(*) means significant

Among aminoglycoside resistance genes, *aac(6ʹ)-lb* gene was the most commonly detected in H1 hospital. However, in H2 hospital, both *aac(6ʹ)-lb* and *aac(3)-II* genes were the most prevalent. Similarly, in H3 hospital, *aac(6ʹ)-lb, aac(3)-II,* and *rmtB* genes were the most commonly detected.

Concerning *mcr-1* colistin resistance gene, it was detected most frequently in H1 hospital, followed by H2 hospital (Table 4).

Statistical analysis showed that there was no significant correlation between the prevalence of resistance genes and the resistance category of the isolates, except for the *aph(3')-I* and *ndm-1* genes, where the prevalence of *aph(3')-I* was higher in XDR (41.5%), compared to MDR (0%) isolates (*P* = 0.004), while *ndm*-*1* was most prevalent among MDR (92.3%) than XDR (33.9%) isolates (*P* = 0.001, Table 4).

### ERIC-PCR typing of *P*. *aeruginosa* clinical isolates

The ERIC-PCR, performed on 70 *P. aeruginosa* isolates showed different DNA fingerprints. The dendrogram map (Fig. 5) revealed 10 different groups, in which 4 isolates had unique ERIC types, while the remaining 66 strains were clustered into six clusters (A-F) based on 80% similarity. Clusters F and A included the largest number of ERIC patterns, 29 (41.4%) and 14 (20%), respectively. However lower percentages could be obtained in clusters B, C and D, with 7 (10%), 6 (8.6%) and 6 (8.6%) patterns, respectively. The lowest percentage was identified in group E, which included only 4 (5.7%) ERIC patterns. Interestingly, it was found that both A and B clusters grouped only XDR isolates collected from H1 hospital. However, cluster C contained only isolates from H2 hospital, while cluster E grouped the isolates from H3 hospital (with similarity > 95%), in addition to one isolate from H2 hospital. Moreover, most of the MDR isolates (84.6%) grouped into cluster F.

### Distribution of the mobile genetic element genes among the isolates

Distribution of the mobile genetic element determinants (*merA, intI, traA,* and *tnp513)* among XDR-PA isolates revealed that *intI* was the most frequently detected gene, as it was amplified in 52 isolates (98.1% of XDR-PA), while *traA* was the least detected as it was amplified in 13 (24.5% of XDR-PA) isolates (Fig. 5). The current study revealed that 98% of XDR *P. aeruginosa* isolates harbored at least one gene associated with movable genetic elements. Among these isolates, 23 (43.4%) contained 2 genes, while 19 (35.8%) and 6 (11.3%) of isolates coharbored 3 and 4 genes, respectively. In contrast, one isolate (H1b11) was found lacking of these genes. Concerning the MDR isolates, *intI* was the most prevalent gene, amplified in 12 MDR isolates (92%). However, *merA* was the least detected, amplified in 7 MDR isolates (53.8%). In addition, all MDR *P. aeruginosa* isolates harbored at least one gene associated with movable genetic elements, where 5 (38.5%) MDR isolates co-harbored 3 genes, 4 (30.85%) MDR isolates co-harbored 4 genes, and 2 (15.4%) MDR isolates co-harbored 2 genes. Regarding the non-MDR isolates, *merA* was the most detected gene, where it was amplified in 4 (100%) isolates, while *traA* was the least detected as it was amplified in only 2 (50%) isolates. Moreover, 2 (50%) non-MDR isolates co-harbored 3 genes, while one (25%) isolate harbored 4 genes and another (25%) isolate harbored 2 genes (Fig. 5).

## Discussion

*P. aeruginosa* is recognized as one of the primary causes of hospital-acquired infections (HAI), including 10 to 15% of HAI worldwide, especially in immunocompromised patients in intensive care units (ICU) [37]. XDR-PA strains represent a significant public health threat globally [1]. In the current study, 53 isolates (75.7%) were classified as extensively drug-resistant *P. aeruginosa* (Fig. 2). Similar findings were recently reported in Egypt, where Abd El-Baky et al., 2020, reported that 87% of *P. aeruginosa* isolates, obtained from different clinical sources were classified as XDR [5]. In the current study, XDR-PA isolates were obtained from three different hospitals in Egypt, located in different areas and cities, suggesting a high prevalence of XDR *P. aeruginosa* isolates in our region. Because of the overuse of antibiotics, XDR-PA has been unfortunately found to spread widely [38]. Susceptibility testing showed a high frequency of resistance against gentamicin (94.29%), ciprofloxacin (92.86%) and levofloxacin (92.86%). In contrast, the lowest frequencies of resistance were observed toward aztreonam (24.29%). However, intermediate levels were detected for fosfomycin (32.86%) and meropenem (55.71%).  These results were concordant with a recent report from Egypt, where El-Far et al., 2021 stated that 97% of *P. aeruginosa* isolates were gentamicin resistant, however only 21.2% were aztreonam resistant [18]. According to Table 2, there are no significant differences in the distribution of XDR, MDR, and non-MDR isolates among different clinical sources. Similarly, Edward et al., 2023 reported also that there was no statistically significant association between the clinical source of isolates and MDR status [39]. In this study, statistical analysis showed that there was a significant difference between the proportion of XDR, compared to MDR and non-MDR in burn specimens from H2 (*P* = 0.036). In addition, according to the results obtained, in H3 hospital, the *P*-value of 0.0001 indicates a significant association between burn specimens and being non-MDR (sensitive), suggesting that burns at H3 are less likely to harbor MDR strains of bacteria. However, due to the small sample size in H3, it is important to interpret these findings with caution, therefore further studies with larger sample sizes are needed to confirm this association (Table 3).

The value of the MARI 0.200 has been applied to differentiate low- and high-risk areas, where antibiotics are abused [40]. In the current study, MAR indices ranged from 0.38 to 0.94 in most of the isolates (Fig. 3), reflecting that a high proportion of the isolates are likely to be from high-risk source and originate from an environment, where several antimicrobials are overused. Similar results were found in a previous study on *P. aeruginosa,* where 91.2% of the isolates had MARI higher than 0.2 [41]. As illustrated in Fig. 3, the predominance of certain resistance profiles was noticeable among the XDR clinical isolates, which may indicate a high rate of microbial dissemination of the same XDR-PA isolates between patients inter- and intra-hospital, suggesting that the incidence of hospital-acquired infections from XDR-PA has unfortunately spread widely.

One of the most efficient drugs for treating serious infections, caused by Gram-negative rods is the class of carbapenems [42]. However, there has been a recent increase in the number of pathogens displaying resistance to carbapenems. The current study revealed that 72.9% of the isolates showed resistance to at least one antipseudomonal carbapenem. Moreover, carbapenem resistance was observed in 75.8% of the overall MDR and XDR isolates (Fig. 3). Similar high resistance rates were also reported in a previous study from Egypt, where 81.8% of the MDR *P. aeruginosa* isolates were carbapenem resistant [18]. Our findings indicate that carbapenemases production played a crucial role in carbapenem resistance in the extensively drug-resistant *P. aeruginosa* isolates. Previous studies from Egypt also reported a high prevalence of carbapenemases production among carbapenem-resistant *P. aeruginosa* [12, 43]. However, these results were higher compared with another study conducted in China indicated that 35.1% of XDR-PA isolates were positive for MBL and MHT [1], while another study from Iran reported that only 20% of *P. aeruginosa* isolates were ESBLs producers [44]. Therefore, the development of carbapenemases-producing XDR-PA becomes a public health problem in our region leaving few therapeutic choices. In this study, 96.2% of XDR *P. aeruginosa* isolates harbored carbapenemase encoding genes, especially *oxa* and *vim-1 *(Table 4, Fig. 1A Supp.). However, Shaaban et al., 2017 previously reported that both *vim-1* and *vim-2* were amplified in 75% of the carbapenem resistant *P. aeruginosa* isolates, while *ndm-1* in 50% of isolates [15]. On the other hand, different prevalence percentages were reported in a previous study from Egypt, where *ndm-1* was the most frequently detected gene among the XDR-PA isolates, while *vim-1* gene was only detected in 18.1% of isolates [43]. Interestingly, the coexistence of more than one carbapenemase gene is detected in 84.9% of the XDR-PA isolates in the current study (Fig. 5), as previously reported in previous studies in Egypt and worldwide [1, 15, 45, 46].

Aminoglycosides are broad-spectrum antibiotics that are highly effective against aerobic and facultative anaerobic Gram-negative bacteria. They mainly prevent protein synthesis and disrupt the cell membrane [47]. In our study, a high prevalence of aminoglycosides resistance was detected, as 94.3% of isolates showed resistance to one or more tested aminoglycosides antibiotics. In addition, all MDR and XDR isolates (100%) showed resistance to at least one aminoglycoside (Fig. 3). A similar resistance percentage was also found in a previous study conducted in Egypt, where El-Far et al., 2021 found that 97% of *P. aeruginosa* isolates were aminoglycoside resistant [18]. In contrast, a lower resistance percentage (43%) has been found in a previous study from Iran [48].The prevalence of resistance to aminoglycosides varies through countries for a variety of reasons, including abuse of these medications in hospitals, geographical, cultural differences and arbitrary use of the antibiotics by people without a prescription [48]. The production of aminoglycoside-modifying enzymes, in addition to16S rRNA methyltransferases are considered the primary causes of bacterial resistance to aminoglycosides. The current study showed that 95.5% of aminoglycoside-resistant isolates contained at least one AMEs gene (Fig. 5). A previous study reported that 79% of aminoglycoside-resistant *P. aeruginosa* isolates harbored AME-encoding genes [48], whilst El-Far et al., 2021, reported that only 59.4% of the aminoglycoside-resistant isolates possessed resistance genes [18]. Interestingly, the current study showed that 40 XDR-PA (75.5%) isolates carried at least three aminoglycosides-resistance genes. However, contrary findings were reported in another study in Saudi Arabia, where none of the *P. aeruginosa* isolates co-harbored more than one gene of aminoglycoside resistance [49]. The most frequently detectable aminoglycoside resistance gene was *aac(6ʹ)-lb*, followed by *aac(3)-II* and *rmtB,* which encodes 16S rRNA methylase, and their distribution percentages among the XDR-PA clinical isolates were 84.9%, 81%, and 75.5%, respectively (Table 4, Fig. 1B Supp.). Previous studies also reported that *aac(6ʹ)-lb* gene was the most prevalent in *P. aeruginosa* in Venezuela and Iran [37, 48]. Other previous studies reported different prevalence of AMEs genes among *P. aeruginosa* worldwide, where *aac(6´)-II* and *ant(2´´)-*I were the most common aminoglycoside resistance determinants found in Europe, while *aph(3′)-VI, ant(2´´)-I, aac(6´)-I*, mainly detected in Korea. However, *aac(6´)-31*/*aadA1* and *aadA2* were mainly found in Mexico and Brazil [50, 51]. This variation in the distribution of AMEs genes may be explained by the geographic differences, variations in prescription patterns of aminoglycosides antibiotics, or in the selection of bacterial populations [49].

Unfortunately, it has recently been noted that the number of colistin-resistant *P. aeruginosa* strains is increasing. Despite the increasing prevalence of such strains, no efficient commercial antibiotics have been developed yet. Therefore, it is clear that further studies on developing effective therapeutic alternatives are urgently needed [52]. Osei Sekyere, 2019 has reported that the appearance of carbapenem- and colistin-resistant determinants in a single strain marks the beginning of a new era of pandrug resistance [53]. Unfortunately, our study detected a high level of resistance toward colistin sulphate (92.86%) (Fig. 1), which may be attributed to improper use of bactericidal agents in our country's intensive care units and insufficient infection control measures. The medical staff has inevitably reintroduced older antimicrobials such as colistin, which is the last resort for treating MDR and XDR infections leading to the spread of colistin-resistant *P. aeruginosa* [54]. Additionally, similar to other antibiotics, the use of colistin is not just limited to humans, as it has been frequently applied to animals to promote growth and to agriculture to ensure high production, therefore the development of colistin-resistant Gram-negative bacteria has been considerably impacted by this practice [5, 55]. The current study also revealed a high rate of resistance towards Polymyxin B (95.7%) (Fig. 1), whilst the pattern of resistance usually increase by time, as a lower level of resistance was found in an older study in Egypt, where 46% and 28.7% of *P. aeruginosa* isolates were resistant to polymyxin B and colistin sulfate, respectively [56]. Abd El-Baky et al., 2020 also reported that 21.3% of *P. aeruginosa* isolates showed resistance to colistin antibiotic [5]. This varriable sensitivity to colistin in various studies from Egypt might be attributed to differences in the geographic zones or the use of different antibiotic regimens in these areas [57]. Previously, Farhan et al., 2019 has observed that 74.1% of ESBL-producing *P. aeruginosa* were polymyxin B resistant, while 37% were colistin resistant [56]. The current study showed that 97.5% of ESBL-producing XDR-PA exhibited polymyxin B resistance, while 95% were colistin resistant. Plasmid-mediated colistin resistance is associated with the addition of a phosphoethanolamine (PEtN) moiety to the anionic phosphate groups of lipid A; the binding site for polymyxins (colistin and polymyxin B). As a consequence, lipid A has lower anionic charges, which prevents electrostatic interactions with cationic polypeptide molecules such as polymyxins, resulting in antimicrobial resistance. The transferable plasmid-mediated colistin resistance gene, *mcr*, is a phosphoethanolamine transferase enzyme, which transfers phosphoethanolamine to lipid A through the horizontal transfer of colistin resistance [58]. In our study, genetic detection of the colistin resistance gene showed that 9 isolates were positive for *mcr-1* (12.3% of colistin resistant isolates; 12.9% of total isolates) (Table 4, Fig. 1C Supp.). Similar results were also reported in a previous study, where the *mcr-1* gene was amplified in 10% of colistin-resistant *P. aeruginosa* isolates; 1.19% of total isolates [55]. Colistin-resistant isolates lacking *mcr-1* gene might have been subjected to mutations due to the long-term use of antibiotics [5]. Further studies are also needed to investigate the exact mechanism of polymyxins resistance and the prevalence of other *mcr* genes among *P. aeruginosa* isolates.

Plasmids, transposons, insertion sequences and integrons are examples of movable genetic elements that play an important role in the horizontal transfer of resistance genes, thus increasing antibiotic resistance. The most frequent cause of multiple antibiotic resistance (MAR) is the existence of plasmids, which have the ability to carry one or more resistance genes, each of which encodes a specific antibiotic resistance trait [41]. Moreover, according to previous studies, more than 130 unique gene cassettes have been identified in Class 1 integrons, the majority of which encode proteins with resistance to all major antibiotic classes [59, 60]. Among the screened genes (*intI, traA, tnp513, and merA)*, *intI* was the most detected gene as it was amplified in 67 (95.7%) isolates, followed by *tnp513* gene, which was harbored by 47 (67.1%) isolates, and *merA* gene, which was present in 43 (61.4%) isolates (Fig. 1D Supp.). Concerning XDR-PA isolates, *intI* was also the most frequently detected gene, as it was amplified in 52 isolates (98.1% of XDR-PA), followed by *tnp513 and merA,* which were present in 64.2% and 60.4% of XDR-PA isolates, respectively. On the other hand, *traA* was amplified in 24.5% of XDR-PA isolates as shown in Fig. 5. Our observations are in agreement with another published study, where Li et al., 2016 previously reported that *intI, tnp513 and merA* were the most detected genes among XDR *P. aeruginosa* isolates [1]. The current study revealed that 98% of the XDR isolates harbored at least one gene associated with mobile genetic elements, 47% of the XDR-PA clinical isolates co-harbored three or four mobile genetic element genes, in agreement with a previous report [1], which might have aided the evolution of these extensively drug-resistant *P. aeruginosa* strains.

ERIC-PCR is a simple and fast typing method that has been widely used in routine epidemiological studies in *P. aeruginosa*. Based on the dendrogram map, six major clusters, representing 66 isolates, were detected as shown in Fig. 5. It was found that both A and B clusters grouped only isolates collected from H1 hospital, while cluster C contained only isolates from H2 hospital. In contrast, cluster E grouped all isolates from H3 hospital (with similarity > 95%), in addition to one isolate from H2 hospital, collected at the same period of time. However, clusters D and F contained isolates from both H1 and H2 hospitals, indicating microbial dissemination of the same isolates intra-and inter-hospital, as previously reported in other studies [36, 61]. Interestingly, we found that most of the MDR isolates (11/13) grouped into cluster F. Furthermore, clusters A and B were unique for XDR-PA isolates. As previously reported [62, 63], our results suggest that ERIC-PCR typing may be essential in identifying the source of *P. aeruginosa* transmission in hospitals and for regular epidemiological surveillance. Therefore, ERIC-PCR can be used as an ideal screening genotyping technique for *P. aeruginosa*.

## Conclusions

In conclusion, the current study revealed that the clinical XDR-PA isolates were resistant to a variety of antimicrobial agents due to several mechanisms and co-harboring multiple antibiotic resistance genes. Additionally, 98% of the XDR-PA isolates possessed at least one gene associated with mobile genetic elements, which could have facilitated the fast evolution of these XDR-PA strains. The increased rates of antimicrobial resistance and the appearance of XDR-PA hospital-acquired infections are becoming a serious public health threat globally. High rates of multidrug resistance draw attention to the urgent need for widespread, local antimicrobial resistance surveillance and efficient multidrug resistance overcoming strategies. The monitoring of both multiple antibiotic resistance (MAR) and antibiotic consumption particularly in hospital-acquired infections is critical for setting up and audit of such strategies.

### Supplementary Information

Supplementary Material 1.

Supplementary Material 2. Fig. 1 Supp. Agarose gel electrophoresis for detection of rsistance-encoding genes and movablegenetic elements among P. aeruginosaclinical isolates using PCR. Lane M is the molecular weight DNA marker. Lane C is a negative control. A Carbapenemases-encoding genes. B Aminoglycosides-resistance genes. C Colistin-resistance gene. D Genes associated with mavable genetic elements.

## Data Availability

All data generated or analyzed in the present work are included in this article. The datasets analyzed and used during the present work are available from the corresponding author on reasonable request.

## Abbreviations

XDR: Extensively drug-resistant
MDR: Multi-drug resistant
PDR: Pandrug-resistant
MIC: Minimum inhibitory concentration
ICU: Intensive care units
XDR-PA: Extensively drug-resistant *Pseudomonas aeruginosa*
AMEs: Aminoglycoside-modifying enzymes
LB: Luria–Bertani
MARI: Multiple antibiotic resistance index
UPGMA: Unweighted pair group method with arithmetic mean clustering method
MHT: Modified Hodge test
MBL: Metallo β-lactamase
ESBL: Extended-spectrum β-lactamase
ERIC-PCR: Enterobacterial repetitive intergenic consensus polymerase chain reaction
CDT: Combined disk test
