# Supplementary material for: Characterization and genetic analysis of extensively drug-resistant hospital acquired Pseudomonas aeruginosa isolates
Source: BMC Microbiol. 2024 Jun 26;24:225. doi: 10.1186/s12866-024-03321-5 (PMC11201863; doi:10.1186/s12866-024-03321-5)
Supplement: Supplementary file 1 — Supplementary Material 1. [file 12866_2024_3321_MOESM1_ESM.docx]

| **Isolate code** | **Date of collection** | **Clinical source** | **hospital** |
| --- | --- | --- | --- |
| **H1b1**  **Supplementary Table 1**: Clinical data of 70 *Pseudomonas aeruginosa* isolates | 23/11/2019 | Burn | H1 |
| **H1b2** | 23/11/2019 | Burn | H1 |
| **H1b3** | 14/12/2019 | Burn | H1 |
| **H1b4** | 15/12/2019 | Burn | H1 |
| **H1b5** | 28/12/2019 | Burn | H1 |
| **H1b6** | 8/1/2020 | Burn | H1 |
| **H1b7** | 11/1/2020 | Burn | H1 |
| **H1b8** | 13/1/2020 | Burn | H1 |
| **H1b9** | 13/1/2020 | Burn | H1 |
| **H1b10** | 20/1/2020 | Burn | H1 |
| **H1b11** | 29/1/2020 | Burn | H1 |
| **H1b12** | 3/2/2020 | Burn | H1 |
| **H1b13** | 18/2/2020 | Burn | H1 |
| **H1w1** | 16/11/2019 | Wound | H1 |
| **H1w2** | 23/11/2019 | Wound | H1 |
| **H1w3** | 12/12/2019 | Wound | H1 |
| **H1w4** | 15/12/2019 | Wound | H1 |
| **H1w5** | 13/1/2020 | Wound | H1 |
| **H1w6** | 16/1/2020 | Wound | H1 |
| **H1w7** | 18/1/2020 | Wound | H1 |
| **H1w8** | 28/1/2020 | Wound | H1 |
| **H1w9** | 8/2/2020 | Wound | H1 |
| **H1w10** | 11/2/2020 | Wound | H1 |
| **H1w11** | 24/2/2020 | Wound | H1 |
| **H1w12** | 14/12/2019 | Wound | H1 |
| **H1w13** | 15/12/2019 | Wound | H1 |
| **H1w14** | 1/2/2020 | Wound | H1 |
| **H1u1** | 3/11/2019 | Urine | H1 |
| **H1u2** | 11/11/2019 | Urine | H1 |
| **H1u3** | 11/11/2019 | Urine | H1 |
| **H1u4** | 11/11/2019 | Urine | H1 |
| **H1u5** | 23/12/2019 | Urine | H1 |
| **H1u6** | 28/12/2019 | Urine | H1 |
| **H1u7** | 28/12/2019 | Urine | H1 |
| **H1u8** | 28/1/2020 | Urine | H1 |
| **H1u9** | 29/1/2020 | Urine | H1 |
| **H1u10** | 1/2/2020 | Urine | H1 |
| **H1u11** | 1/2/2020 | Urine | H1 |
| **H1u12** | 8/2/2020 | Urine | H1 |
| **H1u13** | 12/2/2020 | Urine | H1 |
| **H1u14** | 15/2/2020 | Urine | H1 |
| **H1u15** | 15/2/2020 | Urine | H1 |
| **H1u16** | 18/2/2020 | Urine | H1 |
| **H1 bl 1** | 7/11/2019 | Blood | H1 |
| **H1 bl 2** | 8/1/2020 | Blood | H1 |
| **H1s1** | 4/11/2019 | Sputum | H1 |
| **H1s2** | 23/12/2019 | Sputum | H1 |
| **H1s3** | 8/1/2020 | Sputum | H1 |
| **H1s4** | 8/1/2020 | Sputum | H1 |
| **H1s5** | 4/2/2020 | Sputum | H1 |
| **H1s6** | 18/2/2020 | Sputum | H1 |
| **H1p1** | 19/2/2020 | Pus | H1 |
| **H2b1** | 26/10/2019 | Burn | H2 |
| **H2b2** | 26/10/2019 | Burn | H2 |
| **H2b3** | 8/12/2019 | Burn | H2 |
| **H2b4** | 8/12/2019 | Burn | H2 |
| **H2b5** | 25/12/2019 | Burn | H2 |
| **H2b6** | 6/1/2020 | Burn | H2 |
| **H2b7** | 6/1/2020 | Burn | H2 |
| **H2b8** | 19/2/2020 | Burn | H2 |
| **H2b9** | 19/2/2020 | Burn | H2 |
| **H2b10** | 19/2/2020 | Burn | H2 |
| **H2b11** | 19/2/2020 | Burn | H2 |
| **H2w1** | 26/10/2019 | Wound | H2 |
| **H2w2** | 24/12/2019 | Wound | H2 |
| **H2w3** | 26/2/2020 | Wound | H2 |
| **H2p1** | 26/10/2019 | Pus | H2 |
| **H3b1** | 22/10/2019 | Burn | H3 |
| **H3b2** | 18/11/2019 | Burn | H3 |
| **H3b3** | 28/12/2019 | Burn | H3 |

**H1: Hospital 1, H2: Hospital 2, H3: Hospital 3, b: burn, w: wound, u: urine, bl: blood, s: sputum, p: pus**

**Isolate code; first letter and number: hospital code, second letter and number: clinical source and isolate number**
