# Supplementary material for: Characterization and genetic analysis of extensively drug-resistant hospital acquired Pseudomonas aeruginosa isolates
Source: BMC Microbiol. 2024 Jun 26;24:225. doi: 10.1186/s12866-024-03321-5 (PMC11201863; doi:10.1186/s12866-024-03321-5)

***Ndm-1* (133 bp) *Vim-1* (920 bp) *Vim-2* (774 bp)  *Oxa*(137 bp)**

**A**

**M H2w3 H1u9 H1u15 H1b1 H1b3 H2p1 H1w3 H1b3 H2b3 H1b10 H1w4 H2p1**


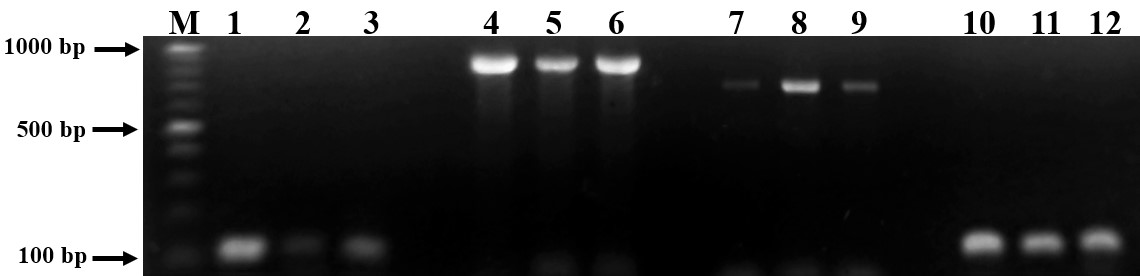


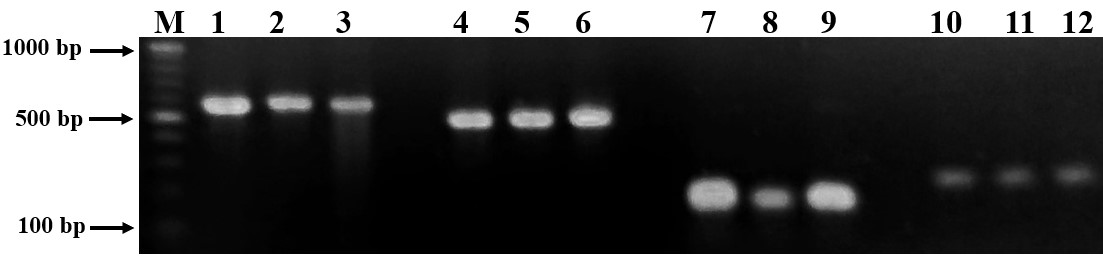


***Aac(3)-II* (567 bp) *Aac(6ʹ)-lb* (481 bp) *RmtB* (173 bp) *Aph(3')-I*(222 bp)**

**B**

**M H1u3 H1b10 H1w3 H1b1 H1b10 H1w4 H1b2 H1b3 H1u3 H1b5 H1u5 H1u16**


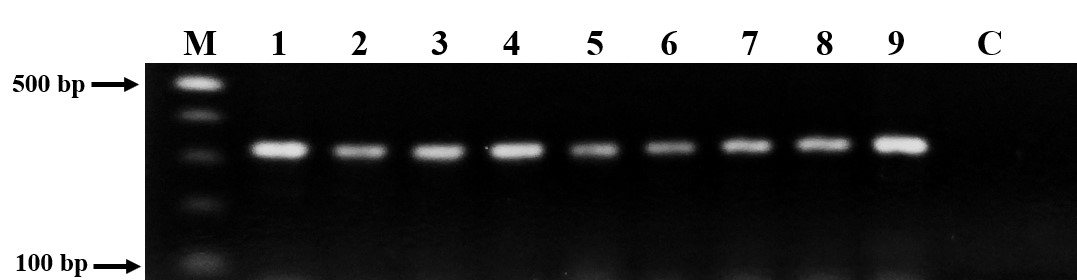

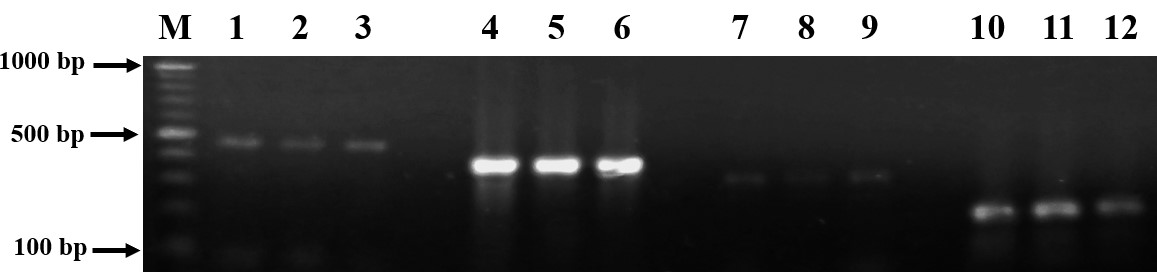


***Mcr-1*(320 bp)**

**M H1b2 H1b5 H1b8 H1b11 H1s6 H1b9 H1w7 H1u16 H2p1**

**Fig.1.**Agarose gel electrophoresis for detection of rsistance-encoding genes and movable [genetic elements](https://academic.oup.com/cid/article-abstract/9/2/357/330758) among *P. aeruginosa* clinical isolates using PCR**. Lane M** is the molecular weight DNA ladder. **Lane C** is a negative control. **A**.Carbapenemases-encoding genes. **B**.Aminoglycosides-resistance genes. **C**. Colistin-resistance gene. **D**. Genes associated with movable genetic elements

**C**

***MerA* (462 bp) *IntI* (373 bp) *TraA* (310 bp) *Tnp513* (200 bp)**

**M H1u16 H1b5 H1w2 H1u10 H1u11 H1u12 H1u10 H1u11 H1u14 H1w13 H1w14 H1u1 H1u**

**D**

**Raw gel images**

**A**


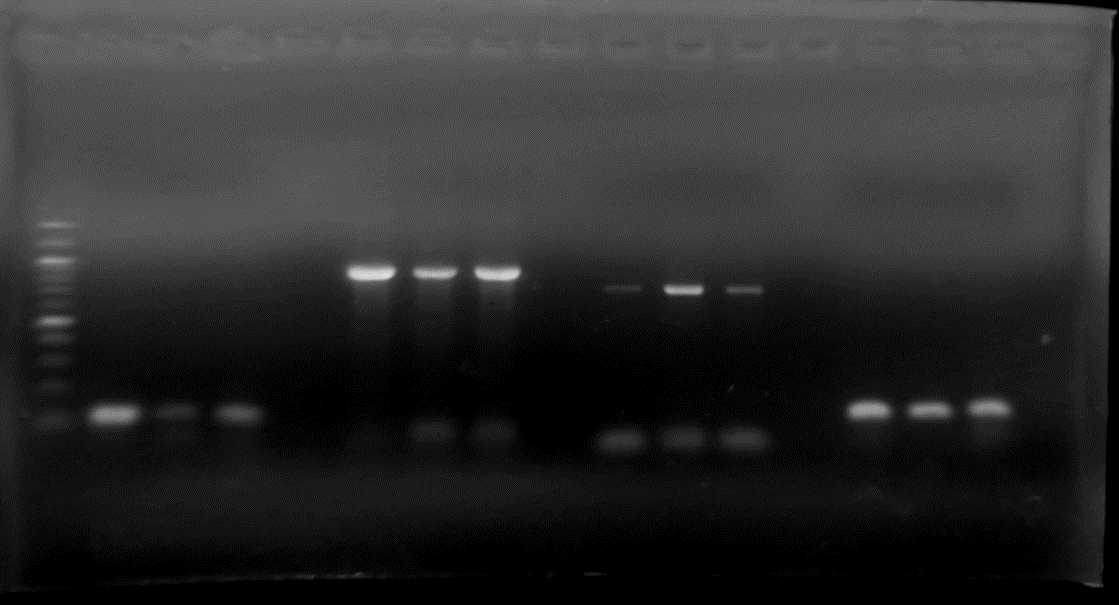


**B**


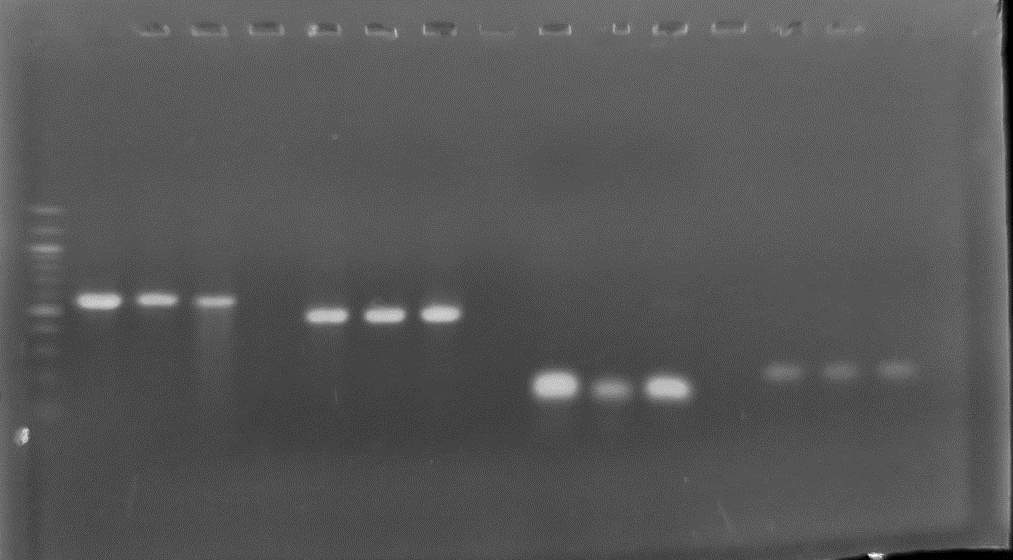


**C**


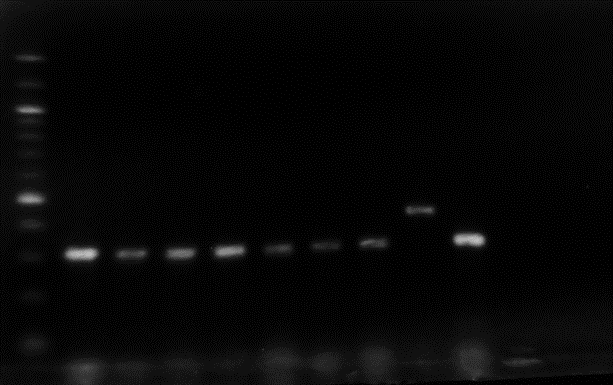


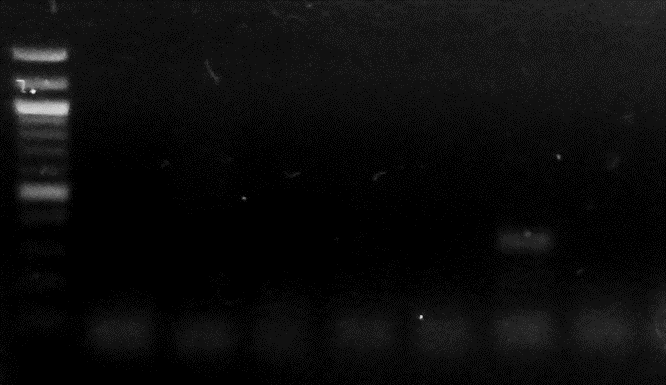


**D**


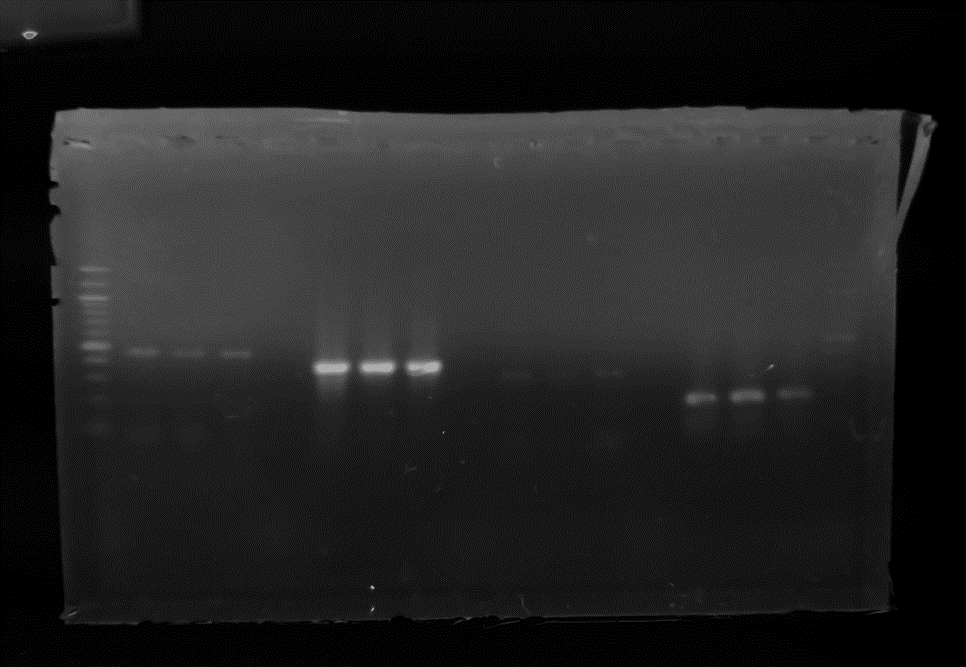

Supplement: Supplementary file 2 — Supplementary Material 2. Fig. 1 Supp. Agarose gel electrophoresis for detection of rsistance-encoding genes and movablegenetic elements among P. aeruginosaclinical isolates using PCR. Lane M is the molecular weight DNA marker. Lane C is a negative control. A Carbapenemases-encoding genes. B Aminoglycosides-resistance genes. C Colistin-resistance gene. D Genes associated with mavable genetic elements. [file 12866_2024_3321_MOESM2_ESM.docx]
